# Clinical importance of TERT overexpression in hepatocellular carcinoma treated with curative surgical resection in HBV endemic area

**DOI:** 10.1038/s41598-017-12469-2

**Published:** 2017-09-25

**Authors:** Jeong Il Yu, Changhoon Choi, Sang Yun Ha, Cheol-Keun Park, So Young Kang, Jae-Won Joh, Seung Woon Paik, Seonwoo Kim, Minji Kim, Sang Hoon Jung, Hee Chul Park

**Affiliations:** 1 Departments of Radiation Oncology, Samsung Medical Center, Sungkyunkwan University School of Medicine, Seoul, Republic of Korea; 2 Pathology, Samsung Medical Center, Sungkyunkwan University School of Medicine, Seoul, Republic of Korea; 3 Surgery, Samsung Medical Center, Sungkyunkwan University School of Medicine, Seoul, Republic of Korea; 4 Medicine, Samsung Medical Center, Sungkyunkwan University School of Medicine, Seoul, Republic of Korea; 5 Statistics and Data Center, Samsung Medical Center, Seoul, Republic of Korea; 6 Department of Medical Device Management and Research, Samsung Advanced Institute for Health Sciences and Technology, Sungkyunkwan University, Seoul, Republic of Korea

## Abstract

This study was designed to investigate the associations between TERT overexpression and the clinicopathologic factors of hepatocellular carcinoma (HCC). A total of 291 patients with HCC were enrolled. The site of first recurrence (anywhere in the liver) was classified as intrahepatic recurrence (IHR). Recurrence was then sub classified as either early or late IHR according to whether it was discovered within 2 years of resection, or after, respectively. TERT overexpression was not significantly correlated with previously recognized prognostic factors. During follow-up, early IHR occurred in 126 (63.6%) patients, while late IHR was detected in 59 patients among 145 patients who remained free of HCC recurrence for ≥ 2 years after surgery. Multivariate analysis showed late IHR was significantly correlated with TERT overexpression (P < 0.001, hazard ratio [HR] 2.67, 95% confidence interval [CI] 1.51–4.72). Intrahepatic metastasis (*P* < 0.001, HR 4.48, 95% CI 2.62–7.65) and TERT overexpression (*P* < 0.001, HR 1.77, 95% CI 1.28–2.45) were significant prognostic factors for IHR-free survival in both univariate and multivariate analyses. TERT overexpression was the only significant prognostic factor for late IHR in HCC treated with curative resection. And, the statistical significance of TERT overexpression on late IHR was limited to HBsAg-positive patients.

## Introduction

Hepatocellular carcinoma (HCC) remains one of the most challenging health problems worldwide despite the hepatitis B virus (HBV) vaccine, surveillance in high-risk patients, potent antiviral agents, and imaging and treatment modalities. Currently, HCC is the fifth most common cancer, and the second leading cause of cancer-related death worldwide^1^.

The Barcelona Clinic Liver Cancer (BCLC) system is the most widely used HCC staging and treatment guideline. According to BCLC, the curative treatments for HCC include liver transplantation (LT), surgical resection, and local ablative therapy, including radiofrequency ablation (RFA)^2^. One study found that the local control rate of HCC was comparable between surgical resection and RFA^3^. Surgical resection is generally preferred, if possible, however, because of the difference in overall recurrence after surgery compared to that after other treatment modalities^4–6^. Unfortunately, recurrence is still detected in approximately 50% of patients after surgical resection^7^. There is no standardized adjuvant treatment modality after HCC surgical resection. Therefore, it is very important to identify reliable biomarkers of recurrence, as well as the most probable sites of recurrence, in order to develop customized management plans.

Telomerase reverse transcriptase (TERT) is a core catalytic component of telomerase that plays a crucial role in maintaining telomere length^8^. In most somatic cells, TERT expression is suppressed. In contrast, TERT is actively expressed in self-renewing cells, such as stem cells^9^. Overexpression of TERT and telomere dysfunction is frequently detected in a variety of human cancer specimens, including thyroid cancer^10^, bladder cancer^11^, melanoma^12^, and brain tumors^13^. This TERT overexpression is detected in up to 90% of cancer cells, compared to in <20% of normal cells^14^. Evidence has suggested that TERT overexpression in HCC is associated with poor clinical outcomes; however, this hypothesis was based on a small patient population, without consideration of other clinicopathologic factors that might affect clinical outcomes^15^. Therefore, it is essential to more precisely evaluate the role of TERT overexpression in HCC, as well as that of other clinicopathologic factors in homogenously-treated patients.

We investigated the associations between TERT overexpression and the clinicopathologic factors of HCC in Korean patients mainly caused by HBV infection. We also studied the clinical significance of TERT overexpression with regard to HCC recurrence and survival duration

## Results

### Patients

The patients and tumor characteristics of the 291 enrolled cases of HCC are displayed in Table 1. The median age was 53 years (range, 17 to 76 years). More than 80% of the patients were male. All but one patient had Child-Pugh class A status. The median alpha-fetoprotein (AFP) level was 169.5 ng/ml (range, 1.0–1667054.0 ng/ml). Approximately one third of the patients (109, 37.5%) were staged as B, while 15 patients (5.2%) were staged as C via the Barcelona Clinic Liver Cancer staging system. According to the Milan criteria, 173 patients were satisfied with the criteria, while 118 patients were not. The median follow-up period was 134.8 months (range, 1.7–193.4 months).

**Table 1:** Patient and tumor characteristics.

Characteristics		No. of patients (%)
Age (years)	Median (range)	53 (17–76)
Sex Etiology	Male	240 (82.5)
	Female	51 (17.5)
	Hepatitis B virus	220 (75.6)
	Hepatitis C virus	30 (10.3)
	Alcohol	18 (6.2)
	Others	23 (7.9)
Child-Pugh class	A	290 (99.7)
	B	1 (0.3)
Albumin	Median (range)	4.0 (2.8–5.0)
	>3.5 g/dL	261 (89.7)
	≤3.5 g/dL	30 (10.3)
Total bilirubin (mg/dL)	Median (range)	0.6 (0.2–1.9)
Initial AFP level (ng/ml)	Median (range)	169.5 (1.0–1667054.0)
	≤200	175 (60.1)
	>200	105 (36.1)
	Not evaluated	11 (3.8)
AJCC T stage, 7^th^ ed.	1	124 (42.6)
	2	116 (39.9)
	3A	33 (11.3)
	3B	12 (4.1)
	4	6 (2.1)
BLCL stage	0	2 (0.7)
	A1	144 (49.5)
	A2	5 (1.7)
	A4	16 (5.5)
	B	109 (37.5)
	C	15 (5.2)
Pathologic tumor size (cm)	Median (range)	3.7 (1.0–21.0)
	<5	193 (66.3)
	≥5	98 (33.7)
Edmonson grade	I	32 (11.0)
	II	235 (80.8)
	III	24 (8.2)
Microvascular invasion	Yes	159 (54.6)
	No	132 (45.4)
Intrahepatic metastasis	Yes	68 (23.4)
	No	223 76.6)
Milan criteria	Within	173 (59.5)
	Beyond	118 (40.5)
Background liver status	LC	146 (50.2)
	Chronic active hepatitis (CAH)	67 (23.0)
	Chronic persistent hepatitis (CPH)	44 (15.1)
	Alcoholic hepatitis	15 (5.2)
	Reactive hepatitis	13 (4.5)
	Others	6 (2.1)

AFP, alpha-fetoprotein; AJCC, American Joint Committee on Cancer; BCLC, Barcelona Clinic Liver Cancer; LC, liver cirrhosis; CAH, chronic active hepatitis; CPH, Chronic persistent hepatitis.

### Correlation between TERT messenger ribonucleic acid (RNA) expression and clinicopathologic variables

**Figure 1:**
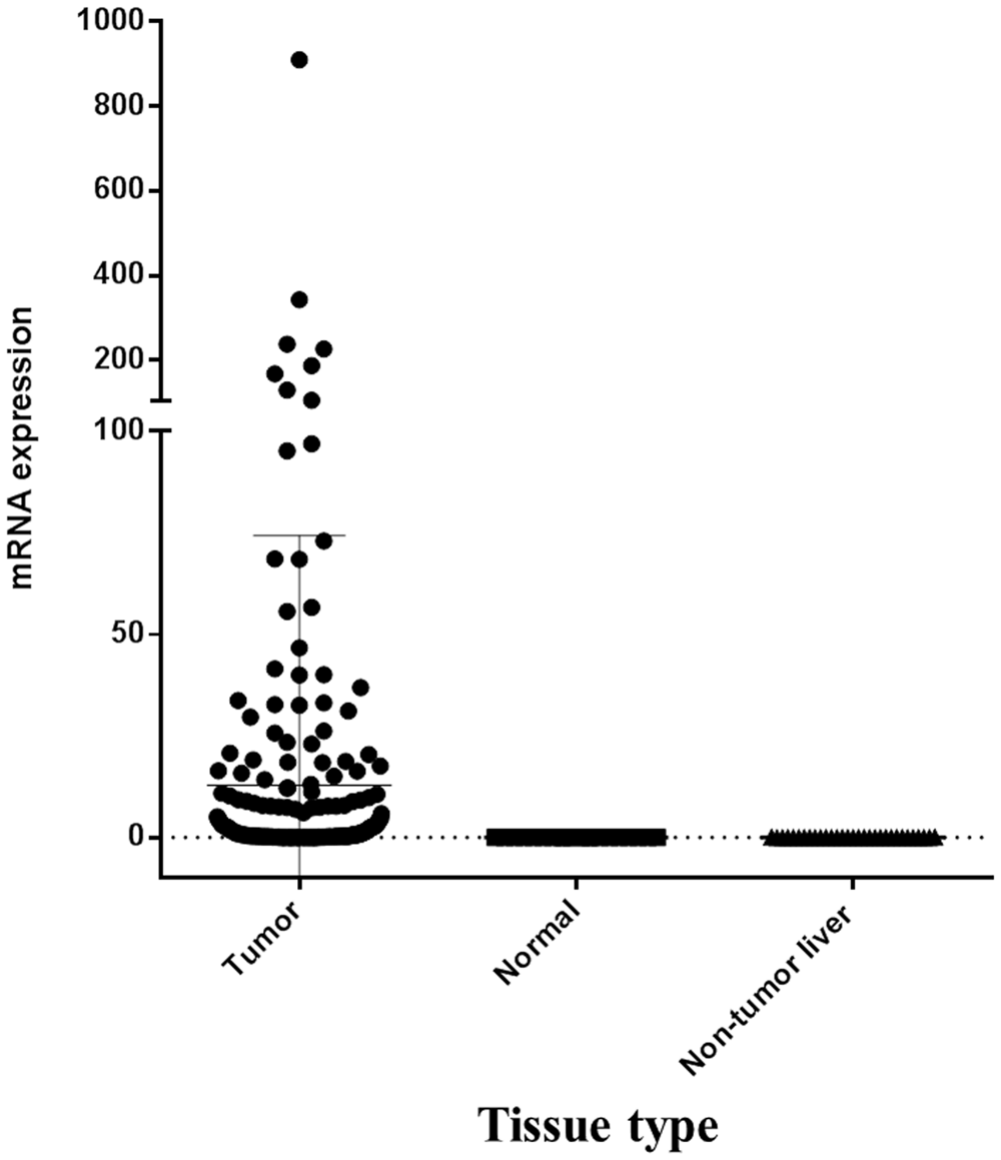
TERT mRNA expression in normal and HCC tissues: TERT mRNA expression (measured using quantitative RT-PCR) was significantly higher in HCC tissues than in adjacent, normal liver tissues (at least 2 cm from the HCC) and in normal liver tissues (obtained from patients after hepatectomy performed for pathology-confirmed metastatic colon cancer of the liver in the absence of viral hepatitis and history of alcohol use).

Gene expression analysis using quantitative Reverse-Transcriptase Polymerase Chain Reaction (qRT-PCR) revealed that TERT messenger RNA (mRNA) expression was significantly higher in HCC tissues than it was in background, non-tumor liver tissue (at least 2 cm from the HCC) and normal liver tissues. Normal liver tissue was obtained from patients who underwent hepatectomy due to pathologically-confirmed metastatic colon cancer to the liver without a history of viral hepatitis or alcohol use (*P* < 0.001, Fig. 1). The median value of normalized TERT mRNA expression values was 0.46 (range, 0.00–908.87). A TERT mRNA value of 0.3 was defined as the cut-off point for high expression of overall recurrence in this present study. This value was based on the highest Youden index values of 1.3147 and 1.3195 with TERT mRNA values of 0.317 and 0.297 (*P*-values 0.0006 for both cut-off points). Diagnostic accuracy of TERT mRNA of 0.3 to predict recurrence and survival was displayed in Supplementary Table 1. And, the sensitivity, specificity, positive predictive value, negative predictive value, positive likelihood ratio, and negative likelihood ratio of TERT mRNA value of 0.3 was 71.3%, 45.6%, 12.6%, 93.5%, 1.31 and 0.63 at 3-year IHR, respectively. For internal validation of the estimated cut-off value of TERT expression, the median of the estimated cut-off values of 1000 bootstrap samples was 0.3 with a 95% confidence interval of 0.3.

The correlations between elevated TERT mRNA expression and clinicopathologic variables are displayed in Supplementary Table 2. High TERT expression was not significantly associated with any other factors, including well-known prognostic factors for HCC recurrence after surgical resection. These factors for HCC recurrence include Edmonson grade (*P* = 0.67), microvascular invasion (*P* = 0.91), intrahepatic metastasis (*P* = 0.40), and background liver status (*P* = 0.89).

### Recurrence patterns and clinicopathologic variables

**Figure 2:**
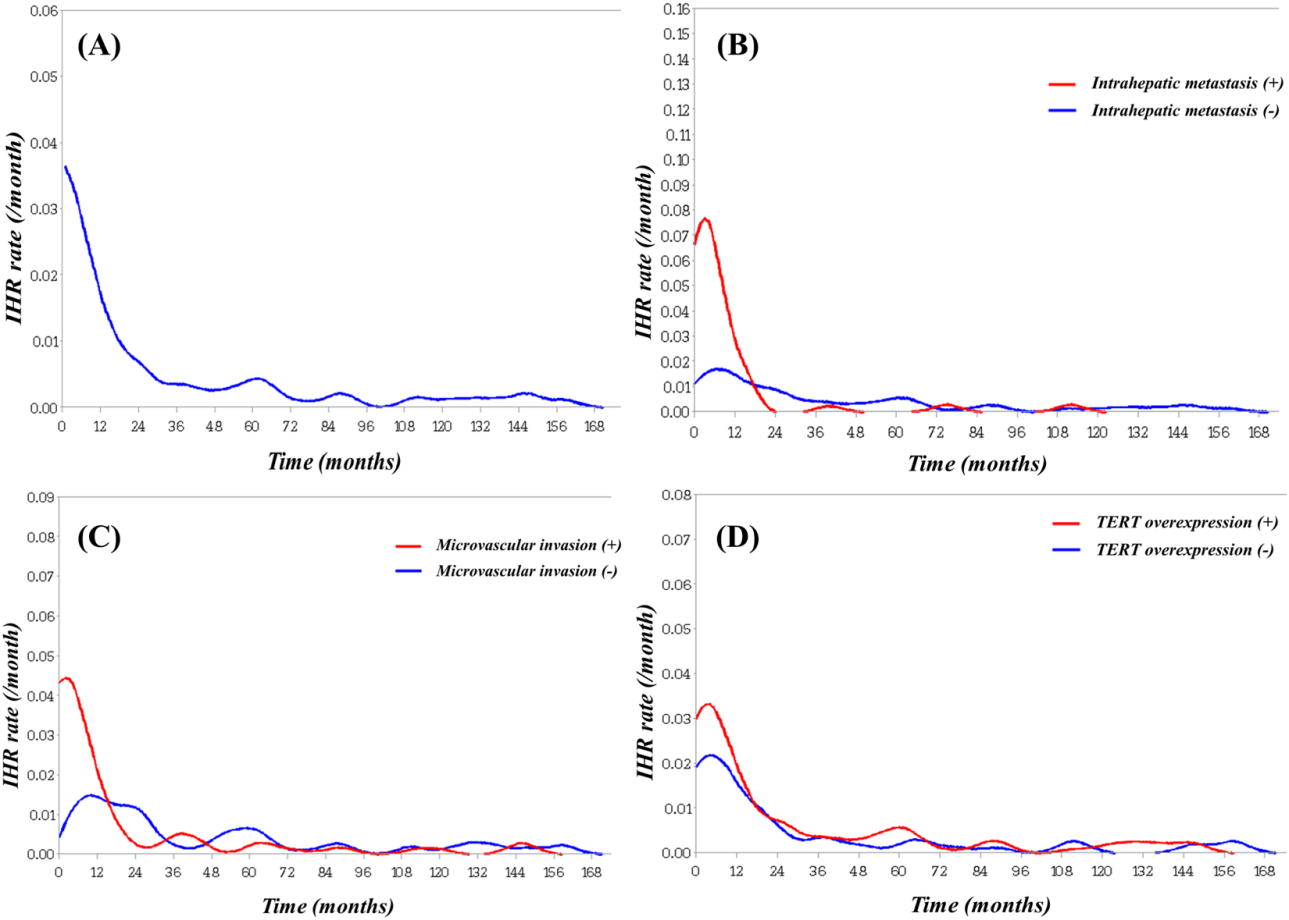
The estimated yearly recurrence rate of HCC after surgical resection: The peak recurrence rate was detected 1-year after resection and maintained, after reaching 0.1 per year (**A**). Distinct from the other curves, curves of TERT overexpression were divided continuously, even after 3 years (**B**).

During the follow-up period, overall recurrence was detected in 198 (68.0%) patients. Among them, 103 (35.4%) patients had isolated intrahepatic recurrence (IHR), 82 (28.2%) had both IHR and distant metastases (DM), and the remaining 13 (4.5%) had isolated DM. Among all of the patients with IHR (with or without DM), early IHR developed in 126 (63.6%) patients. In contrast, late IHR was detected in 59 of the 145 patients that were followed for over 2 years. The estimated recurrence rate (per month) after surgical resection is displayed in Fig. 2A. The highest recurrence rate was detected within 6 months of resection, and decreased gradually thereafter. There was a relatively stable rate of recurrence after 3 years postoperatively, at approximately 0.005% per month.

The results of univariate and multivariate analyses of clinicopathologic variables and early/late IHR are displayed in Tables 2 and 3. Multivariate analysis showed early IHR was significantly correlated with tumors ≥ 5 cm in size (*P* = 0.02, hazard ratio [HR] 2.81, 95% confidence interval [CI] 1.20–6.60), and intrahepatic metastasis (*P* < 0.001, HR 5.27, 95% CI 2.81–9.87). Early IHR was somewhat related to TERT expression, although the trend was not statistically significant (*P* = 0.08, HR 1.41, 95% CI 0.96–2.08).

**Table 2:** Univariate and multivariate analysis of probable prognostic factors in early intrahepatic recurrence-free survival (n = 291).

Variables	Univariate			Multivariate		
	HR	95% CI	*P*	HR	95% CI	*P*
Sex						
Male *vs*. Female	1.12	0.70–1.80	0.63			
Age (years)						
≥55 *vs*. <55	0.96	0.68–1.37	0.82			
HBsAg						
Positive *vs*. Negative	1.56	1.01–2.40	0.04	1.08	0.68–1.74	0.74
Albumin level (dg/dl)						
≤3.5 *vs*. >3.5	2.00	1.21–3.30	0.007	1.06	0.58–1.93	0.86
Initial AFP level (ng/ml)						
>200 *vs*. ≤200 (main effect)	2.28	1.25–4.17	0.008	0.98	0.66–1.45	0.92
> 200 *vs*. ≤200 (time-dependent effect)	0.93	0.84–0.96	0.03			
ALBI grade						
2 *vs*. 1 (main effect)	2.13	1.50–3.03	<0.001	0.88	0.46–1.68	0.69
2 *vs*. 1 (time-dependent effect)				1.07	1.01–1.14	0.02
Tumor size (cm)						
≥ 5 *vs*. < 5 (main effect)	4.49	2.40–8.43	< 0.001	2.81	1.20–6.60	0.02
≥5 *vs*. <5 (time-dependent effect)	0.89	0.83–0.96	0.001	0.89	0.83–0.96	0.002
Edmonson grade						
III *vs*. I or II	1.76	1.01–3.06	0.05	1.01	0.55–1.87	0.96
Microvascular invasion						
Yes *vs*. No (main effect)	8.31	3.82–18.08	<0.001			
Yes *vs*. No (time-dependent effect)	0.89	0.83–0.95	<0.001	1.39	0.83–2.34	0.21
Intrahepatic metastasis						
Yes *vs*. No	6.01	4.17–8.66	<0.001	5.27	2.81–9.87	<0.001
Milan criteria						
Beyond *vs*. within	1.20	0.84–1.70	0.32			
AJCC T stage						
3 or 4 *vs*. 1 or 2	4.03	2.75–5.93	<0.001	0.74	0.33–1.68	0.47
BCLC stage^+^						
B or C *vs*. 0 or A (main effect)	6.81	3.46–13.40	<0.001			
B or C *vs*. 0 or A (time- dependent effect)	0.89	0.84–0.96	<0.001			
Background liver status						
LC or CAH *vs*. others	1.93	1.22–3.06	0.005	1.45	0.87–2.40	0.15
*TERT* overexpression						
Yes vs. No	1.45	1.00–2.09	0.05	1.41	0.96–2.08	0.08

IHR, intrahepatic recurrence; AFP, alpha-fetoprotein; AJCC, American Joint Committee on Cancer; BCLC, Barcelona Clinic Liver Cancer; LC, liver cirrhosis; CAH, chronic active hepatitis; CPH, Chronic persistent hepatitis. Univariate analysis: Cox Proportional Hazard model or time-dependent Cox model. Multivariate analysis: time-dependent Cox model. ^+^Not included in multivariable analysis due to multicollinearity with tumor size.

**Table 3:** Univariate and multivariate analysis of probable prognostic factors in late intrahepatic recurrence-free survival (n = 145).

Variables	Univariate			Multivariate		
	HR	95% CI	*P*	HR	95% CI	*P*
Sex						
Male *vs*. Female	1.2	0.61–2.36	0.6			
Age (years)						
≥55 *vs*. <55 (main effect)	0.24	0.07–0.76	0.02	1.21	0.71–2.05	0.49
≥55 *vs*. <55 (time-dependent effect)	1.02	1.01–1.04	0.005			
HBsAg						
Positive *vs*. Negative	1.85	0.96–3.55	0.07	2.27	1.16–4.46	0.02
Albumin level (g/dl)						
≤3.5 *vs*. >3.5	1.78	0.64–4.93	0.27			
Initial AFP level (ng/ml)						
>200 *vs*. ≤200	1.52	0.89–2.57	0.12			
ALBI grade						
2 *vs*. 1	1.27	0.68–2.34	0.46			
Tumor size (cm)						
≥5 *vs*. <5	0.74	0.39–1.39	0.35			
Edmonson grade						
III *vs*. I or II	0.93	0.29–2.98	0.9			
Microvascular invasion						
Yes *vs*. No	0.91	0.54–1.54	0.72			
Intrahepatic metastasis						
Yes *vs*. No	1.21	0.38–3.87	0.75			
Milan criteria						
Beyond *vs*. within	1.11	0.66–1.87	0.68			
AJCC T stage						
3 or 4 *vs*. 1 or 2	1.01	0.32–3.24	0.98			
BCLC stage						
B or C *vs*. 0 or A	0.74	0.40–1.36	0.33			
Background liver status						
LC or CAH *vs*. others	1.37	0.76–2.47	0.29			
*TERT* overexpression						
Yes *vs*. No	2.42	1.38–4.26	0.002	2.67	1.51–4.72	<0.001

In contrast, late IHR was not significantly correlated with any variables, including those known to be significant prognostic factors of HCC, including microvascular invasion (*P* = 0.72), intrahepatic metastasis (*P* = 0.75), and BCLC stage (*P* = 0.33). Multivariate analysis showed TERT expression (*P* < 0.001, HR 2.67, 95% CI 1.51–4.72) and HBsAg (*P* = 0.02, HR 2.27, 95% CI 1.16–4.46) were the only significant prognostic factors for late IHR. In the subgroup analysis by HBsAg status, late IHR was significantly associated with TERT overexpression in HBsAg-positive patients (*P* = 0.002, HR 2.77, 95% CI 1.44–5.34), but not in HBsAg-negative patients (*P* = 0.22, HR 2.70, 95% CI 0.55–13.25). The monthly IHR rate according to probable prognostic clinicopathologic factors is displayed in Fig. 2B–D. Even after two years, the IHR curve for TERT overexpression was maintained at a higher level compared to that of non-TERT overexpression.

### Survival outcomes and probable prognostic factors

Table 4 summarizes the univariate analysis results of overall IHR-free survival (IHRFS), DM-free survival (DMFS), and recurrence-free survival (RFS) according to probable prognostic factors.

**Table 4:** Univariate analysis of probable prognostic factors in intrahepatic recurrence-free survival (IHRFS), distant metastasis-free survival (DMFS) and recurrence-free survival (RFS).

Variables	IHRFS			DMFS			RFS		
	HR	95% CI	*P*	HR	95% CI	*P*	HR	95% CI	*P*
Sex									
Female *vs*. Male	1.15	0.78–1.69	0.49	0.8	0.50–1.29	0.35	1.05	0.73–1.52	0.78
Age (years)									
≤55 *vs*. >55	1	0.74–1.32	0.93	0.95	0.63–1.42	0.79	0.9	0.68–1.20	0.48
HBsAg									
Positive *vs*. Negative	1.64	1.15–2.36	0.007	1.63	0.97–2.76	0.07	1.63	1.16–2.30	0.005
Albumin level (g/dl)									
≤3.5 *vs*. >3.5	1.96	1.25–3.06	0.003	1.73	0.94–3.17	0.08	1.86	1.20–2.89	0.005
ALBI grade									
2 *vs*. 1	1.85	1.37–2.50	<0.001	1.59	1.05–2.42	0.03	1.87	1.40–2.50	<0.001
Initial AFP level (ng/ml)									
>200 *vs*. ≤200	1.38	1.02–1.86	0.03	1.75	1.16–2.64	0.07	1.58	1.18–2.10	0.002
Tumor size (cm)									
≥5 *vs*. <5 (main effect)	1.93	1.31–2.85	<0.001	4.3	2.38–7.76	<0.001	2.3	1.59–3.32	<0.001
≥5 *vs*. <5 (time-dependent effect)	0.99	0.98–1.00	0.03	0.98	0.97–1.00	0.01	0.99	0.98–1.00	0.02
Edmonson grade									
III *vs*. I or II	1.51	0.92–2.50	0.1	2.91	1.64–5.14	<0.001	1.82	1.13–2.92	0.01
Microvascular invasion									
Yes *vs*. No (main effect)	2.83	1.90–4.22	<0.001	4.91	2.50–9.65	<0.001	3.26	2.21–4.81	<0.001
Yes *vs*. No (time-dependent effect)	0.99	0.98–1.00	0.003	0.99	0.98–1.00	0.03	0.99	0.98–0.99	0.002
Intrahepatic metastasis									
Yes *vs*. No (main effect)	6.68	4.32–10.33	<0.001	4.81	3.15–7.36	<0.001	6.74	4.40–10.30	<0.001
Yes *vs*. No (time-dependent effect)	0.98	0.96–1.00	0.03				0.97	0.95–1.00	0.03
Milan criteria									
Beyond *vs*. within	1.17	0.87–1.57	0.29	1.31	0.87–1.96	0.19	1.19	0.90–1.58	0.22
AJCC T stage									
3 or 4 *vs*. 1 or 2 (main effect)	3.22	2.27–4.58	<0.001	7.55	4.10–13.93	<0.001	3.85	2.75–5.41	<0.001
3 or 4 *vs*. 1 or 2 (time-dependent effect)				0.98	0.97–1.00	0.04			
BCLC stage									
B or C *vs*. 0 or A (main effect)	2.87	1.93–4.25	<0.001	2.78	1.85–4.18	<0.001	3.2	2.20–4.65	<0.001
B or C *vs*. 0 or A (time-dependent effect)	0.98	0.97–0.99	0.002				0.98	0.97–0.99	0.001
Background liver status									
LC or CAH *vs*. others	1.71	1.19–2.45	0.004	1.17	0.73–1.86	0.52	1.65	1.17–2.32	0.005
*TERT* overexpression									
Yes *vs*. No	1.7	1.25–2.31	<0.001	0.94	0.63–1.42	0.77	1.55	1.16–2.08	0.003

AFP, alpha-fetoprotein; AJCC, American Joint Committee on Cancer; BCLC, Barcelona Clinic Liver Cancer; LC, liver cirrhosis; CAH, chronic active hepatitis.

**Figure 3:**
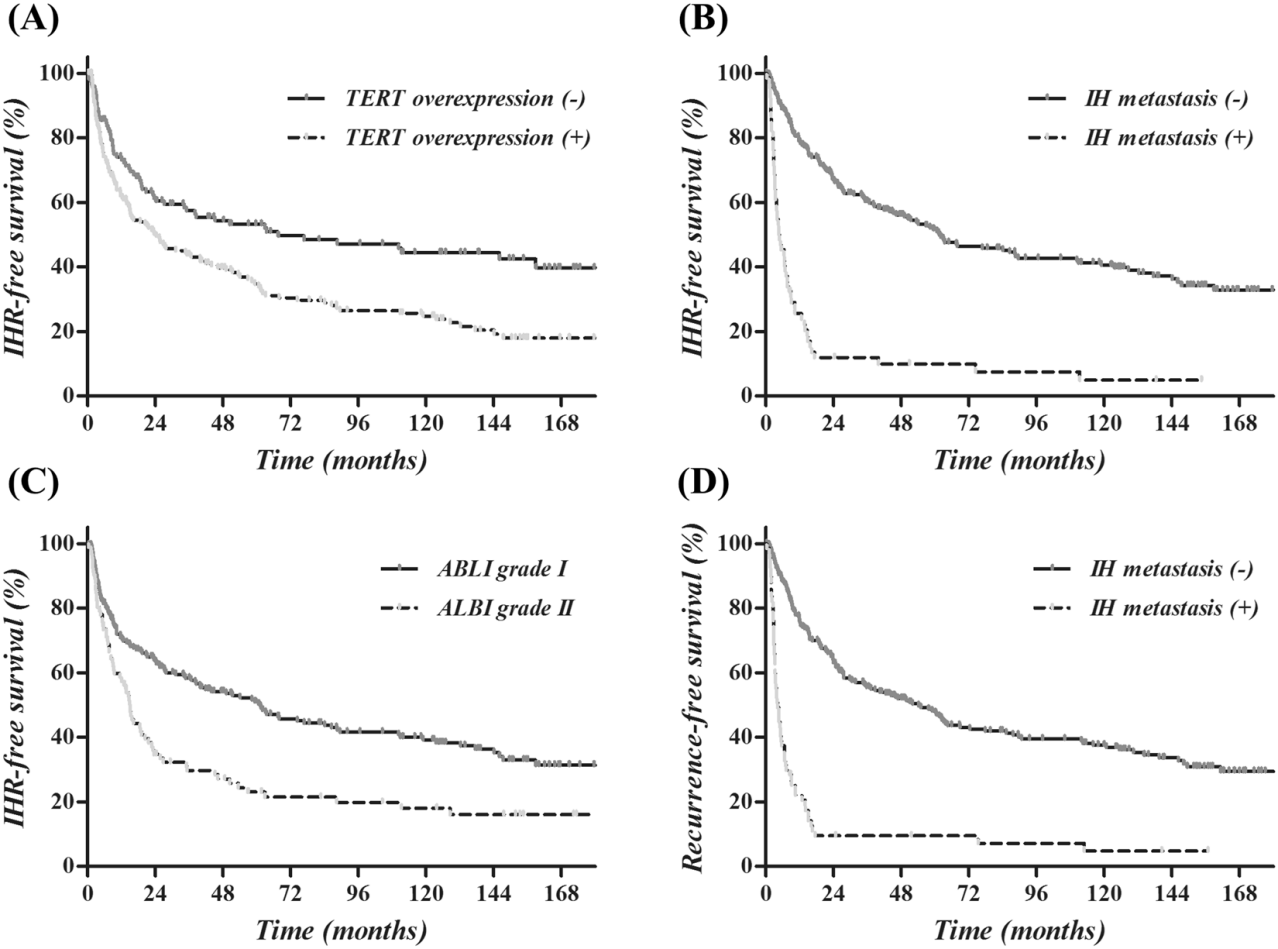
Kaplan-Meier survival curves according to probable prognostic factors: TERT overexpression (**A**) and intrahepatic metastasis (**B**) were significant prognostic factors of IHRFS and ALBI grade (**C**) was marginally significant on multivariate analysis. Intrahepatic metastasis (**D**) was also a significant prognostic factor for RFS.

Multivariate analysis (Table 5) showed statistical significance only for TERT overexpression (*P* < 0.001, HR 1.77, 95% CI 1.28–2.45, Fig. 3A) and intrahepatic metastasis (*P* < 0.001, HR 4.48, 95% CI 2.62–7.65, Fig. 3B) on overall IHRFS. ALBI grade was marginally significant (*P* = 0.05, HR 1.42, 95% CI 0.99–2.04, Fig. 3C). In the DMFS, intrahepatic metastasis (*P* = 0.02, HR 2.50, 95% CI 1.14–5.47, Supplementary Fig. 1A) was the only significant prognostic factor, while Edmonson grade was marginally significant (*P* = 0.06, HR 1.79, 95% CI 0.97–3.30, Supplementary Fig. 1B). In addition, DMFS did not vary according to TERT expression (*P* = 0.86, Supplementary Fig. 1C). Finally, RFS was significantly affected by intrahepatic metastasis (*P* < 0.001, HR 3.33, 95% CI 1.93–5.77, Fig. 1D) and marginally by ALBI grade (*P* = 0.06, HR 1.39, 95% CI 0.99–1.97, Fig. 3D). TERT expression did not affect RFS (*P* = 0.56, HR 1.12, 95% CI 0.77–1.65); however, the effect of TERT expression (*P* = 0.005, HR 1.01, 95% CI 1.00–1.02) on RFS increased with time.

**Table 5:** Multivariate analysis of probable prognostic factors in intrahepatic recurrence-free survival (IHRFS),distant metastasis-free survival (DMFS) and recurrence-free survival (RFS).

Variables	IHRFS			DMFS			RFS		
	HR	95% CI	*P*	HR	95% CI	*P*	HR	95% CI	*P*
HBsAg									
Positive *vs*. Negative	1.19	0.80–1.76	0.39	1.46	0.82–2.60	0.2	1.25	0.86–1.82	0.25
Albumin level (g/dl)									
≤3.5 *vs*. >3.5	1.19	0.71–2.00	0.51	1.51	0.74–3.07	0.26	1.23	0.74–2.04	0.14
ALBI grade									
2 *vs*. 1	1.42	0.99–2.04	0.05	1.27	0.78–2.07	0.34	1.39	0.99–1.97	0.06
Initial AFP level (ng/ml)									
>200 *vs*. ≤200	1.07	0.77–1.47	0.7	1.07	0.68–1.68	0.78	1.14	0.83–1.56	0.41
Tumor size (cm)									
≥5 *vs*. <5	0.93	0.58–1.47	0.74	1.16	0.62–2.17	0.65	0.99	0.63–1.56	0.98
Edmonson grade									
III *vs*. I or II				1.79	0.97–3.30	0.06	1.04	0.62–1.75	0.88
Microvascular invasion									
Yes *vs*. No	1.13	0.77–1.66	0.54	1.52	0.86–2.70	0.15	1.28	0.88–1.89	0.2
Intrahepatic metastasis									
Yes *vs*. No	4.48	2.62–7.65	<0.001	2.5	1.14–5.47	0.02	3.33	1.93–5.77	<0.001
AJCC T stage									
3 or 4 *vs*. 1 or 2	0.9	0.47–1.74	0.75	1.4	0.55–3.58	0.48	1.21	0.62–2.37	0.58
Background liver status									
LC or CAH *vs*. others	1.26	0.84–1.89	0.26				1.34	0.91–1.97	0.14
*TERT* overexpression									
Yes *vs*. No (main effect)	1.77	1.28-2.45	<0.001				1.12	0.77–1.65	0.56
Yes *vs*. No (time-dependent effect)							1.01	1.00–1.02	0.005

AFP, alpha-fetoprotein; AJCC, American Joint Committee on Cancer; BCLC, Barcelona Clinic Liver Cancer; LC, liver cirrhosis; CAH, chronic active hepatitis.

**Figure 4:**
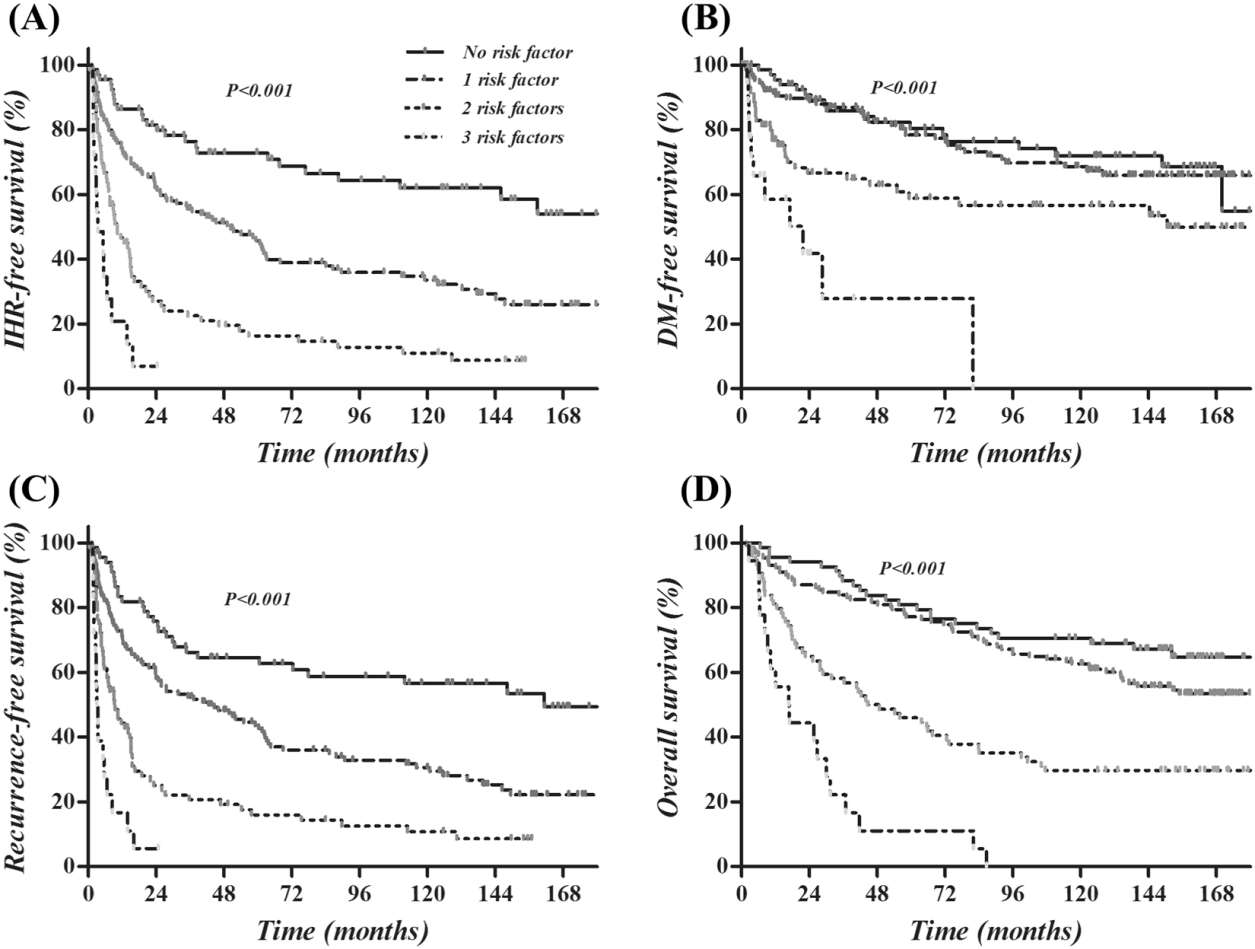
Kaplan-Meier survival curves according to risk grouping: The IHRFS (**A**), DMFS (**B**), RFS (**C**), and OS (**D**) curves according to the grouping using intrahepatic metastasis, ALBI grade, and TERT overexpression are displayed.

We performed predictive grouping of IHRFS according to the significant or marginally significant factors (ALBI grade, intrahepatic metastasis and TERT overexpression) using the following criteria: group 1, no risk factor; group 2, one risk factor; group 3, two risk factors; and group 4, all three risk factors. The curves for IHRFS, DMFS, RFS, and overall survival (OS) according to the risk groupings are shown in Fig. 4. Although the survival curves are significantly different according to group (all *P* < 0.001), an even clearer stratification was identified for IHRFS and RFS. According to the risk grouping, the median IHRFS was not reached in group 1, was 50.3 months (95% CI, 29.4–71.3 months) in group 2, 10.3 months (95% CI, 5.5–15.1 months) in group 3, and 3.4 months in group 4 (95% CI, 0.3–6.4 months). Similarly, RFS was not reached in group 1, but was 42.5 months (95% CI, 19.6–65.4 months) in group 2, 9.4 months (95% CI, 4.9–13.9 months) in group 3, and 3.41 months in group 4 (95% CI, 2.1–4.1 months).

## Discussion

In this study, we evaluated the correlation between clinicopathologic variables and the prognostic significance of TERT overexpression in HCC treated with curative surgical resection. Interestingly, TERT overexpression was not related to other clinicopathologic variables, including well-known prognostic factors for HCC. Instead, TERT overexpression was significantly correlated with late IHR (occurring two years or later after surgical resection). The previously identified prognostic factors for HCC were only related to early IHR in our study. Furthermore, the prognostic significance of TERT overexpression on overall IHRFS was maintained in multivariate analysis.

The TERT is the most important unit of the telomerase complex, which allows potentially unlimited cell proliferation through the addition of repeated TTAGGG sequences and subsequent telomere maintenance^16^. In most normal somatic cells, TERT expression is suppressed and strictly controlled by transcriptional regulation; in some self-renewing cells such as stem cells and/or reproductive cells, however, TERT is expressed more ubiquitously^9^. There has been a lot of interest in understanding the pivotal role of increased TERT-mediated telomere maintenance in HCC carcinogenesis^17^. The mechanisms by which TERT expression is increased in HCC may include TERT gene amplification^18^, and TERT promoter mutations^19,20^.

In addition to HCC carcinogenesis^17^, TERT abnormalities also have prognostic significance in thyroid cancer^10^, bladder cancer^21^, and brain tumors^13^. In HCC, however, there are few studies defining the role of TERT expression as a prognosticator for the disease. It also remains to be determined whether TERT expression during recurrence reflects metastasis or a new primary lesion. Therefore, understanding the crucial role of TERT expression in HCC carcinogenesis^17^, as well as its clinical significance on metastasis and/or de novo primary HCC is extremely important for optimizing HCC management.

This study reveals the prognostic significance of TERT expression on IHRFS. With the exception of other potential confounders during multivariate analysis, TERT expression demonstrated an HR of 1.78 on overall IHRFS. Although the HR of TERT expression on IHRFS was lower than that of intrahepatic metastasis (HR 4.22), it still suggests that it is an independent prognostic factor for overall IHRFS. There was no statistically significant correlation between intrahepatic metastasis and TERT expression (Supplementary Table 2).

With the exception of HBsAg status, TERT was the only significant prognostic factor for late IHR. As displayed in Fig. 3, most cases of IHR developed within 2 years of initial resection in patients with intrahepatic metastasis. In those with TERT expression, more than half of the IHR cases were detected more than 2 years after initial resection. This finding suggests that TERT expression is more closely related to de novo primary HCC, rather than recurrence, in patients initially treated with curative surgical resection.

The subgroup analysis by HBsAg status revealed that a statistically significant association between late IHR and TERT overexpression was limited to HBsAg-positive patients. The most frequent mechanism by which HBV infection leads to TERT overexpression is related to insertional mutagenesis by the virus itself rather than promoter mutations or gene amplification, which is seen in other liver diseases including HCV infection^18,20,22,23^. Integration of HBV was seen in 22% of the HBV positive patients whereas gene amplification was seen in 6.7%^18^. Although it is assumed that HBV integration would be a major cause of TERT overexpression in our samples, we cannot rule out the possibility of other genetic alterations that are involved in the TERT overexpression. Future work is needed to uncover the molecular mechanism underlying the overexpression of TERT and the correlation with late IHR.

Prior research has found that TERT expression induced by TERT promoter mutations is one of the earliest genetic events identified in pre-neoplastic lesions in cirrhotic livers^17^. Therefore, high TERT expression in resected HCC might reflect potential cancer development in the surrounding liver. If this were true, then one could hypothesize that there would be a higher incidence of de novo primary HCC lesions in patients who had TERT expression after hepatic resection. Like other studies in which genetic factors were associated with late tumor recurrence in HCC^24,25^, TERT expression was significantly correlated with late IHR in this study. Patients with high TERT expression, therefore, may require more intensive follow-up after surgical resection. In addition, considering that DMFS was unaffected by TERT expression, it may be important to consider LT, rather than liver resection, in order to improve clinical outcomes in these patients. Further evaluation is needed to clarify the clinical significance of TERT expression in the patients treated with LT.

The role of ALBI grade as a prognosticator for OS is well recognized in HCC, including in patients treated with curative surgical resection^26–29^. Although one group found a slightly higher tendency of recurrence in ALBI grade II than in grade I, some have suggested that ALBI grade is not a significant prognostic factor for HCC recurrence after curative liver resection^26^. Interestingly, however, ALBI grade itself had a marginally significant role as a prognostic factor in this study. There was a marginal survival difference between RFS and IHRFS according to ALBI grade. Correlation analysis showed ALBI grade was significantly related to background liver status and marginally related to HBsAg status (Supplementary Table 3). Therefore, ALBI grade could predict IHR by reflecting the status of the surrounding liver. Regardless, a larger study is needed to evaluate the role of the ALBI grade as a prognostic factor for HCC recurrence.

This study has several limitations that warrant consideration. First, we evaluated the expression of TERT mRNA, but not of TERT promoter mutations and telomere lengthening. However, there is a well-recognized correlation between TERT promotor mutations, TERT expression, and telomere lengthening in other studies. Second, this study has unavoidable selection bias given that it was a single institution, retrospective study. Third, diagnostic performance of TERT mRNA value of 0.3 was not sufficiently high as 61.9 to 71.3% of sensitivity and it has never been validated by independent external data. Finally, we only included patients who had undergone curative surgical resection for HCC. Therefore, our results ought to be interpreted with caution, as they may not be generalizable to HCC patients treated with other modalities. To apply the outcomes of the present study in clinical practice, it is essential to reproduce the results through prospectively designed independent multicenter studies. Future research with larger, prospective studies is needed to substantiate the clinical significance of TERT expression in HCC recurrence.

Nevertheless, this study provides valuable information that is relevant for further study and consequent modifications of HCC management. TERT expression was a significant prognostic factor for IHRFS and RFS. In addition, TERT expression was the only significant factor for late IHR in patients treated with curative hepatic resection. Therefore, close follow-up and/or treatment modifications for resectable HCC according to TERT expression might improve clinical outcomes in these patients.

## Conclusions

In this study conducted in the HBV endemic area, TERT overexpression was not related to previously known HCC prognostic factors treated with curative surgical resection. Known HCC prognostic factors were correlated with early IHR, while TERT overexpression was the only significant prognostic factor for late IHR. Besides TERT overexpression, ALBI grade and presence of intrahepatic metastasis also had prognostic significance in IHRFS and RFS. Further research is needed to substantiate these findings.

## Materials and Methods

### Patients

Cancer tissue from patients with histologically confirmed HCC at Samsung Medical Center (Seoul, Korea) between July 2000 and May 2006 was used in this present study. Patients were eligible if they underwent complete surgical resection with curative intent. Complete resection was defined as the removal of all hepatic tumor nodules with microscopic clear resection margins. Any patients who received neoadjuvant or adjuvant treatments were excluded. The Institutional Review Board at Samsung Medical Center approved this study, and waived informed consent.

Prior to surgery, patients underwent a work-up of their liver function and HCC status. This examination included the following: comprehensive history, patient demographics, viral marker, chest X-ray, complete blood count, chemistry profile, liver function test, indocyanin green retention test, AFP level, and contrast enhanced liver CT and/or magnetic resonance imaging (MRI) of the abdomen and pelvis. Chest CT and whole body bone scan were performed to rule out distant metastasis. Positron emission tomography (PET)-CT was alternatively used for the same purpose at the physician’s discretion.

Patients were generally followed every two to three months after surgical resection. During the follow-up period, evaluations included physical examinations, laboratory tests (including liver function tests and AFP), chest X-rays, and CT of the abdomen and pelvis. Patients with suspicious imaging findings and/or continuously elevated AFP levels were further evaluated with PET-CT and/or MRI.

Based on the Liver Cancer Study Group of Japan guidelines, the following histological parameters were postoperatively assessed: largest tumor diameter, number of tumors, microvascular invasion, and intrahepatic metastasis^16^. The resected HCC tissues were graded according to the Edmonson and Steiner grading system: grade I (well differentiated), grade II (moderately differentiated), or grade III (poorly differentiated)^30^.

### RNA extraction and qRT-PCR

The total RNA was isolated from fresh samples using the ReliaPrep^TM^ FFPE Total RNA Miniprep System (Promega, Madison, WI). RNA was reverse transcribed using a high-capacity, complementary deoxyrinuleic acid (cDNA) Reverse Transcription Kit (Life Technologies, Invitrogen, Carlsbad, CA) according to the manufacturer’s instructions. The expression of TERT was measured using a gene expression assay containing forward and reverse primers, and a FAM-labeled MGB TaqMan probe from Applied Biosystems (assay ID; Hs00972650_m1, Thermo-Fisher Scientific, Waltham, MA) for the detection of overlap at the exon 3-exon 4 junctions. GAPDH was used as an endogenous control (assay ID; Hs99999905_m1, Thermo-Fisher Scientific). The PCR reaction mixture contained TaqMan Universal PCR master mix with AmpErase UNG (Applied Biosystems, Foster City, CA), 900-nM primers (forward and reverse), 250 nM TaqMan probe, and 5 μl of cDNA sample for a total reaction volume of 20 μl. The PCR conditions were as follows: 95 °C for 10 min followed by 40 cycles of amplification at 95 °C for 15 s, and 60 °C for 1 min using the ABI PRISM 7500HT Fast Real-time PCR system (Applied Biosystems). The threshold cycle (C_t_), which is the fractional cycle number at which the amount of amplified target reached a fixed threshold, was determined. The relative changes in gene expression were measured using the 2^−ΔΔCt^ (ΔΔC_t_ = ΔC_t_target gene − ΔC_t_GAPDH) method.

### Definition of recurrence and statistical analysis

Recurrence was generally diagnosed using radiologic examinations without histologic confirmation, except in patients for whom further surgical resection or liver transplantation was indicated. The site of first recurrence was classified as IHR, which is defined as recurrence occurring anywhere inside the entire liver. The IHR was sub classified as either early recurrence or late recurrence according to whether it was detected <2 years or ≥2 years after the initial surgery, respectively. This cut-off point was based on the study by Imamura *et al*.^31^. All other sites of recurrence were defined as DM.

The duration of IHRFS, DMFS, RFS, and OS were calculated from the date of surgical resection to the date of each event, or the last day of follow-up.

The cut-off value of TERT mRNA expression on overall recurrence was determined using the log-rank test and the minimum p-value approach. For internal validation of the estimated cut-off value of TERT expression level, the distribution of the cut-off values was examined using 1000 bootstrap samples with replacement from the study data. *P*-values < 0.05 were considered statistically significant in two-tailed tests. The correlation between high TERT expression or intrahepatic metastasis and other clinicopathologic variables was evaluated using the Chi-square or Fisher’s exact tests. Univariate analysis was performed using the Cox proportional hazard model or the time-dependent Cox model according to satisfaction of the proportional hazard assumption for each variable. The proportional hazard assumption was confirmed using the correlation between partial residuals from the estimated Cox proportional hazard model and the time to the event. Variables with *P* < 0.1 in univariate analysis were included in the multivariate analysis. Multivariate analysis was conducted using a time-dependent Cox model, because some variables included in the multivariate analysis were against the proportional hazard assumption. Multicollinearity was identified by a Variance Inflation Factor (VIF) > 4.0. Some of those variables were excluded from the multivariate analysis. Statistical analyses were performed using SAS version 9.4 (SAS Institute, Cary, NC). Variable risk was expressed as an HR with corresponding 95% CI.

## Electronic supplementary material

Supplementary file
