# Supplementary material for: Clinical importance of TERT overexpression in hepatocellular carcinoma treated with curative surgical resection in HBV endemic area
Source: Sci Rep. 2017 Sep 25;7:12258. doi: 10.1038/s41598-017-12469-2 (PMC5612986; doi:10.1038/s41598-017-12469-2)

**Article type: Original article**

**Clinical importance of TERT overexpression in hepatocellular carcinoma treated with curative surgical resection in HBV endemic area**

Jeong Il Yu^1^, Changhoon Choi^1^, Sang Yun Ha^2^, Cheol-Keun Park^2^, So Young Kang^2^, Jae-Won Joh^3^, Seung Woon Paik^4^, Seonwoo Kim^5^, Minji Kim^5^, Sang Hoon Jung^1^, and Hee Chul Park^1,6^

Departments of ^1^Radiation Oncology, ^2^Pathology, ^3^Surgery, ^4^Medicine, Samsung Medical Center, Sungkyunkwan University School of Medicine, Seoul, Republic of Korea

^5^Statistics and Data Center, Samsung Medical Center, Seoul, Republic of Korea

^6^Department of Medical Device Management and Research, Samsung Advanced Institute for Health Sciences and Technology, Sungkyunkwan University, Seoul, Republic of Korea

Jeong Il Yu and Changhoon Choi contributed equally to this work as first authors.

Hee Chul Park and Cheol-Keun Park contributed equally to this work as corresponding authors.

Running title: **TERT overexpression in HCC**

Address for correspondence: Prof. Hee Chul Park

Department of Radiation Oncology

Samsung Medical Center, Sungkyunkwan University School of Medicine

81 Irwon-ro, Gangnam-gu, Seoul, 06351, Korea

TEL: +82-2-3410-2616, FAX: +82-2-3410-2619

E-mail: hee.ro.park@samsung.com

Address for correspondence: Cheol-Keun Park MD, PhD

Department of Pathology

Samsung Medical Center, Sungkyunkwan University School of Medicine

81 Irwon-ro, Gangnam-gu, 06351, Seoul, Korea

TEL: +82-2-3410-2616, FAX: +82-2-3410-2619

E-mail: [ckpark@skku.edu](mailto:ckpark@skku.edu)

**Supplementary Tables**

**Supplementary Table 1.** Diagnostic accuracy of TERT mRNA of 0.3 to predict recurrence and survival

|  |  | AUC of time dependent ROC curve | | | |  | diagnostic performance of cutoff value of 0.3 (positive if >0.3) | | | | | |
| --- | --- | --- | --- | --- | --- | --- | --- | --- | --- | --- | --- | --- |
| Event | time | AUC (%) | standard error | 95% CI lower | 95% CI upper |  | Sensitivity (%) | Specificity (%) | PPV (%) | NPV (%) | LR positive | LR negative |
| Early IHR | t=12 | 51.5 | 3.6 | 44.5 | 58.6 |  | 67.7 | 45.4 | 37.9 | 74.1 | 1.2394 | 0.7116 |
| Late IHR | t=36 | 64.5 | 8.3 | 48.2 | 80.7 |  | 71.3 | 45.6 | 12.6 | 93.5 | 1.3114 | 0.6285 |
| DMFS | t=12 | 50.4 | 4.9 | 40.7 | 60.0 |  | 63.1 | 41.7 | 14.6 | 87.8 | 1.0836 | 0.8833 |
| DMFS | t=24 | 52.1 | 4.5 | 43.3 | 60.9 |  | 64.8 | 41.6 | 20.4 | 83.7 | 1.1094 | 0.8463 |
| DMFS | t=36 | 51.9 | 4.3 | 43.4 | 60.4 |  | 61.9 | 41.4 | 22.5 | 79.9 | 1.0568 | 0.9196 |
| RFS | t=12 | 50.6 | 3.5 | 43.7 | 57.5 |  | 65.4 | 45.2 | 40.8 | 69.4 | 1.1929 | 0.7662 |
| RFS | t=24 | 51.3 | 3.5 | 44.5 | 58.1 |  | 62.5 | 44.9 | 52.5 | 55.2 | 1.1351 | 0.8344 |
| RFS | t=36 | 52.5 | 3.6 | 45.6 | 59.5 |  | 62.5 | 44.9 | 58.4 | 49.2 | 1.1349 | 0.8346 |

AUC, area under curve; ROC, receiver operating characteristics; CI, confidence interval; PPV, positive predictive value; NPV, negative predictive value; LR, likelihood ratio; IHR, intrahepatic recurrence; DMFS, distant metastasis-free survival; RFS, recurrence-free survival

**Supplementary Table 2.** Correlation between high levels of *TERT* mRNA expression and the clinicopathologic characteristics

|  |  | High level of *TERT* expression | |  |
| --- | --- | --- | --- | --- |
| Variable | No of patients | Positive (%) | Negative (%) | *P* value |
| Sex |  |  |  |  |
| Male  Female | 240  51 | 142 (59.2)  29 (56.9) | 98 (40.8)  22 (43.1) | 0.44 |
| Age (years) |  |  |  |  |
| <55  ≥55 | 163  128 | 100 (61.3)  71 (55.5) | 63 (38.7)  57 (44.5) | 0.19 |
| HBs Ag |  |  |  |  |
| Positive  Negative | 215  76 | 126 (58.6)  45 (59.2) | 89 (41.4)  31 (40.8) | 1.00 |
| Albumin level (g/dl) |  |  |  |  |
| >3.5  ≤3.5 | 261  30 | 149 (57.1)  22 (73.3) | 112 (42.9)  8 (26.7) | 0.12 |
| ALBI grade |  |  |  |  |
| 1  2 | 197  94 | 116 (58.9)  55 (58.5) | 81 (41.1)  39 (41.5) | 1.00 |
| Initial AFP level (ng/ml) |  |  |  |  |
| >200  ≤200 | 105  175 | 65 (61.9)  99 (56.6) | 40 (38.1)  76 (43.4) | 0.45 |
| Tumor size (cm) |  |  |  |  |
| <5  ≥5 | 193  98 | 114 (59.1)  57 (58.2) | 79 (40.9)  41 (41.8) | 0.90 |
| Edmondson grade |  |  |  |  |
| I or II  III | 267  24 | 158 (59.2)  13 (54.2) | 109 (40.8)  11 (45.8) | 0.67 |
| Microvascular invasion |  |  |  |  |
| Yes  No | 159  132 | 94 (59.1)  77 (58.3) | 65 (40.9)  55 (41.7) | 0.91 |
| Intrahepatic metastasis |  |  |  |  |
| Yes  No | 68  223 | 43 (63.2)  128 (57.4) | 25 (36.8)  95 (42.6) | 0.40 |
| Milan criteria |  |  |  |  |
| within  beyond | 173  118 | 106 (61.3)  65 (55.1) | 67 (38.7)  53 (44.9) | 0.33 |
| AJCC T stage |  |  |  |  |
| 1 or 2  3 or 4 | 240  51 | 137 (57.1)  34 (66.7) | 103 (42.9)  17 (33.3) | 0.22 |
| BCLC stage |  |  |  |  |
| 0-A  B-C | 167  124 | 98 (58.7)  73 (58.9) | 69 (41.3)  51 (41.1) | 1.00 |
| Background liver status |  |  |  |  |
| LC or CAH  Other | 213  78 | 126 (59.2)  45 (57.7) | 87 (40.8)  33 (42.3) | 0.89 |

AFP, alph-fetoprotein; AJCC, American Joint Committee on Cancer; BCLC, Barcelona Clinic Liver Cancer; LC, liver cirrhosis; CAH, chronic active hepatitis

**Supplementary Table 3.** Correlation between intrahepatic metastasis and other clinicopathologic characteristics

|  |  | Intrahepatic metastasis | |  |
| --- | --- | --- | --- | --- |
| Variable | No of patients | Positive (%) | Negative (%) | P value |
| Sex |  |  |  |  |
| Male  Female | 240  51 | 55 (22.9)  13 (25.5) | 185 (77.1)  38 (74.5) | 0.72 |
| Age (years) |  |  |  |  |
| <55  ≥55 | 163  128 | 45 (27.6)  23 (18.0) | 118 (72.4)  105 (82.0) | 0.07 |
| HBs Ag |  |  |  |  |
| Positive  Negative | 215  76 | 58 (27.0)  10 (13.2) | 157 (73.0)  66 (86.8) | 0.02 |
| Albumin level (g/dl) |  |  |  |  |
| >3.5  ≤3.5 | 261  30 | 58 (22.2)  10 (33.3) | 203 (77.8)  20 (66.7) | 0.18 |
| ALBI grade |  |  |  |  |
| 1  2 | 197  94 | 38 (19.3)  30 (31.9) | 159 (80.7)  64 (68.1) | 0.03 |
| Initial AFP level (ng/ml) |  |  |  |  |
| >200  ≤200 | 105  175 | 40 (38.1)  26 (14.9) | 65 (61.9)  149 (85.1) | <0.001 |
| Tumor size (cm) |  |  |  |  |
| <5  ≥5 | 193  98 | 23 (11.9)  45 (45.9) | 170 (88.1)  53 (54.1) | <0.001 |
| Edmondson grade |  |  |  |  |
| I or II  III | 267  24 | 57 (21.3)  11 (45.8) | 210 (78.7)  13 (54.2) | 0.01 |
| Microvascular invasion |  |  |  |  |
| Yes  No | 159  132 | 68 (42.8)  0 (0.0) | 91 (57.2)  132 (100.0) | <0.001 |
| Milan criteria |  |  |  |  |
| within  beyond | 173  118 | 33 (19.1)  35 (29.7) | 140 (80.9)  83 (70.3) | 0.05 |
| AJCC T stage |  |  |  |  |
| 1 or 2  3 or 4 | 240  51 | 21 (8.8)  47 (92.2) | 219 (91.3)  4 (7.8) | <0.001 |
| BCLC stage |  |  |  |  |
| 0-A  B-C | 167  124 | 6 (3.6)  62 (50.0) | 161 (96.4)  62 (50.0) | <0.001 |
| Background liver status |  |  |  |  |
| LC or CAH  Other | 213  78 | 51 (23.9)  17 (21.8) | 162 (76.1)  61 (78.2) | 0.76 |

**Supplementary Table 4.** Correlation between ALBI grade and other clinicopathologic characteristics

|  |  | ALBI grade | |  |
| --- | --- | --- | --- | --- |
| Variable | No of patients | 1 (%) | 2 (%) | *P* value |
| Gender |  |  |  |  |
| Male  Female | 240  51 | 164 (68.3)  33 (64.7) | 76 (31.7)  18 (35.3) | 0.62 |
| Age (years) |  |  |  |  |
| <55  ≥55 | 163  128 | 113 (69.3)  84 (65.6) | 50 (30.7)  44 (34.4) | 0.53 |
| HBs Ag |  |  |  |  |
| Positive  Negative | 215  76 | 139 (64.7)  58 (76.3) | 76 (35.3)  18 (23.7) | 0.07 |
| Initial AFP level (ng/ml) |  |  |  |  |
| >200  ≤200 | 105  175 | 71 (67.6)  116 (66.3) | 34 (32.4)  59 (33.7) | 0.90 |
| Tumor size (cm) |  |  |  |  |
| <5  ≥5 | 193  98 | 133 (68.9)  64 (65.3) | 60 (31.1)  34 (34.7) | 0.60 |
| Edmondson grade |  |  |  |  |
| I or II  III | 267  24 | 181 (67.8)  16 (66.7) | 86 (32.2)  8 (33.3) | 1.00 |
| Microvascular invasion |  |  |  |  |
| Yes  No | 159  132 | 106 (66.7)  91 (68.9) | 53 (33.3)  41 (31.1) | 0.71 |
| Milan criteria |  |  |  |  |
| within  beyond | 173  118 | 117 (67.6)  80 (67.8) | 56 (32.4)  38 (32.2) | 1.00 |
| AJCC T stage |  |  |  |  |
| 1 or 2  3 or 4 | 240  51 | 166 (69.2)  31 (60.8) | 74 (30.8)  20 (39.2) | 0.25 |
| BCLC stage |  |  |  |  |
| 0-A  B-C | 167  124 | 120 (71.9)  77 (62.1) | 47 (28.1)  47 (37.9) | 0.10 |
| Background liver status |  |  |  |  |
| LC or CAH  Other | 213  78 | 129 (60.6)  68 (87.2) | 84 (39.4)  10 (12.8) | <0.001 |

**Supplementary Figure Legends**

**Supplementary Fig. 1.** **Kaplan-Meier survival curves according to the probable prognostic factors**: The DMFS curves are stratified by the grouping. Intrahepatic metastasis was a significant prognostic factor (a), and Edmonson grade was marginally significant (b) in multivariate analysis. TERT was not a significant prognostic factor of DMFS (c).

**a**


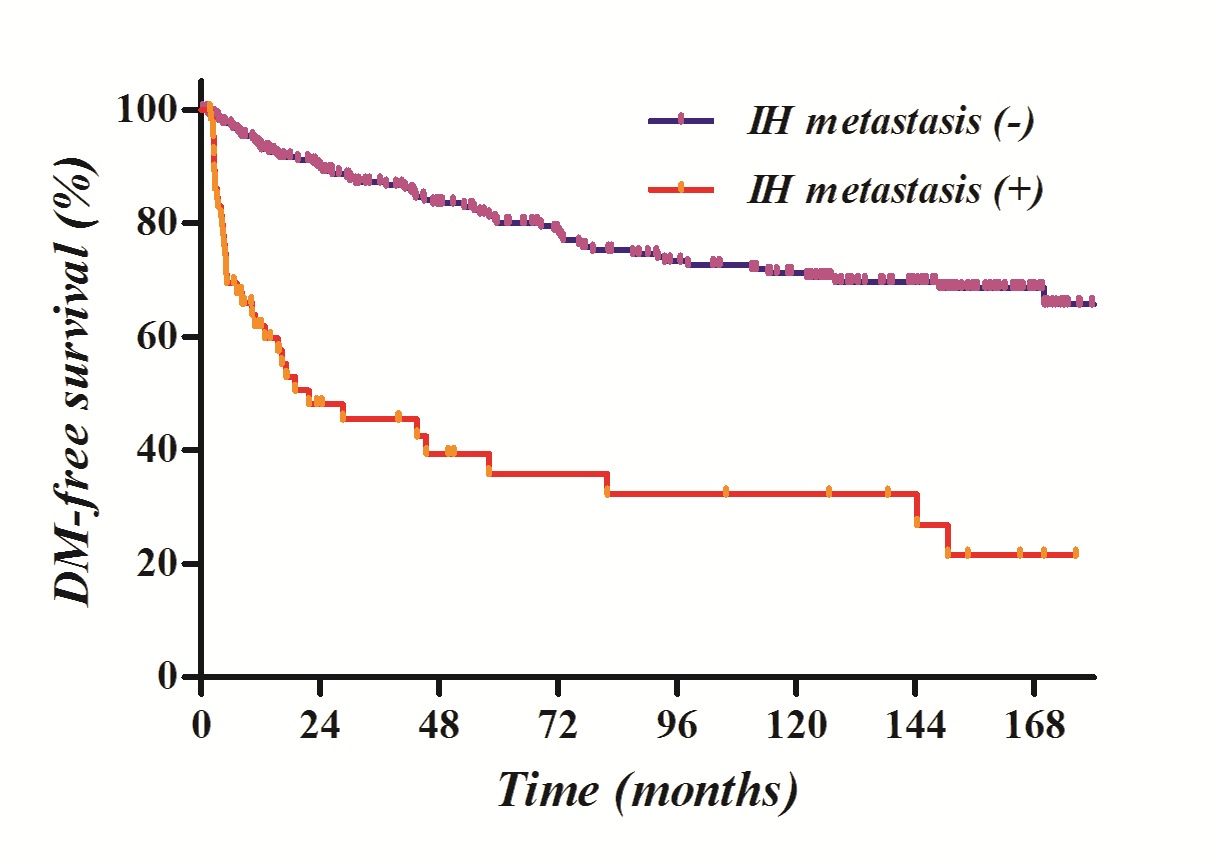


**b**


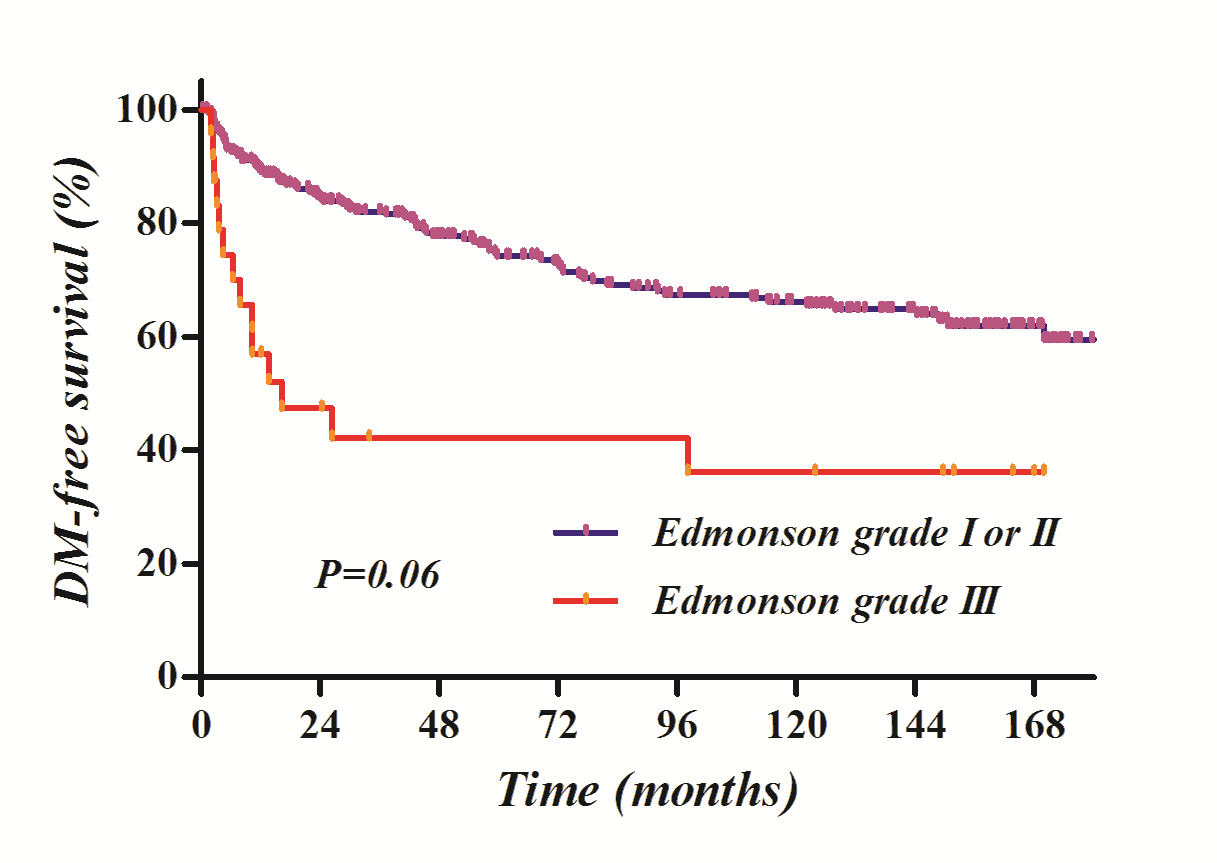


**C**


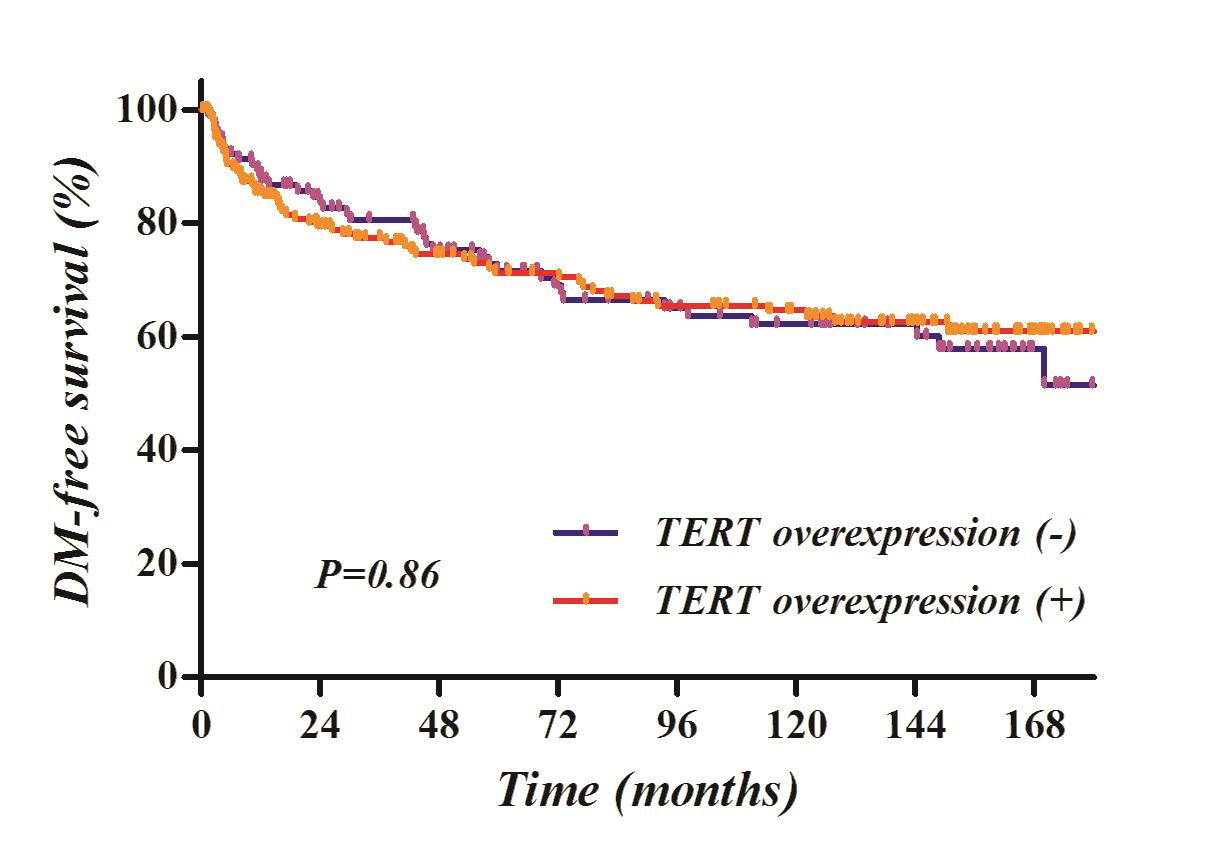

Supplement: Supplementary file 1 — Supplementary file [file 41598_2017_12469_MOESM1_ESM.doc]
